# Mechanism of Apigenin against breast cancer stem cells: network pharmacology and experimental validation

**DOI:** 10.3389/fphar.2024.1496664

**Published:** 2024-11-13

**Authors:** Mengdie Ou, Zhicheng Deng, Yonghui Shi, Jianxiong He, Zicong Ye, Ming Guo, Guohua Cheng, Junyan Wu, Li Lv

**Affiliations:** ^1^ School of Pharmacy, Jinan University, Guangzhou, China; ^2^ Department of Pharmacy, Guangdong Provincial Key Laboratory of Malignant Tumor Epigenetics and Gene Regulation, Sun Yat-Sen Memorial Hospital, Sun Yat-Sen University, Guangzhou, China; ^3^ Guangdong Provincial Key Laboratory of Cancer Pathogenesis and Precision Diagnosis and Treatment, Shenshan Medical Center, Sun Yat-sen Memorial Hospital, Sun Yat-sen University, Shanwei, China

**Keywords:** Apigenin, breast cancer stem cells, MCF-7 cells, PI3K/AKT, p53, network pharmacology, molecular docking

## Abstract

Apigenin (API), a traditionally sourced flavonoid, is recognized for its anti-neoplastic properties. Despite well-documented effects on tumorigenesis, the detailed therapeutic impact on breast cancer stem cells (BCSCs) and the associated molecular mechanisms are yet to be clarified. The objective of this study is to elucidate the therapeutic effects of API on BCSCs and to uncover its molecular mechanisms through network pharmacology and experimental validation. Interactions of API with candidate targets were examined through target screening, enrichment analysis, construction of protein-protein interaction networks, and molecular docking. MCF-7-derived BCSCs were utilized as a model system to investigate and substantiate the anti-BCSC effects of API and the underlying mechanism. Molecular docking studies have shown that API and TP53 exhibit favorable binding affinity. Compared with the negative control group, API effectively suppressed the expression of BCSC-related proteins such as ALDH1A1, NANOG, EpCAM, and MYC, downregulated p-PI3K and p-AKT, and upregulated p53. This study demonstrates that API can play an anti-BCSC role by regulating the PI3K/AKT/p53 pathway in BCSCs of MCF-7 cells, highlighting its potential as a therapeutic agent for targeting BCSCs.

## 1 Introduction

The breast cancer, a malignancy that has seen an alarming increase over the last four decades, is the second most common and most lethal cancer among women worldwide (Giaquinto et al., 2022). The intricate pathogenesis and heterogeneity of breast cancer present significant challenges, even in the context of today’s advanced medical technologies. Despite a range of available drug treatments, the poor prognosis and the incidence of adverse drug reactions continue to impede progress in combating this formidable disease. Emerging research has identified a remarkable subclass of malignant cells within tumors, known as cancer stem cells (CSCs). These cells are considered to play a pivotal role in tumor initiation, recurrence, and resistance to standard therapeutic approaches. Breast cancer stem cells (BCSCs) contribute to the progression and drug resistance of breast cancer through various mechanisms. These mechanisms encompass altering the tumor microenvironment (TME), promoting immune evasion, reshaping metabolic pathways, and activating specific signaling pathways. For example, EMSY transcriptional repressor, BRCA2 interacting protein supports the self-renewal of BCSCs by influencing methionine metabolism (Liu et al., 2024), while aldehyde dehydrogenase 1 family, member a1 (ALDH1A1) activates the transforming growth factor-beta activated kinase 1 - nuclear factor kappa-light-chain-enhancer of activated B cells (TAK1-NFkB) signaling pathway by reducing intracellular pH, increasing granulocyte-macrophage colony-stimulating factor (GM-CSF) secretion, inducing the expansion of immunosuppressive cells, and thereby promoting tumor development (Liu et al., 2021). BCSCs interact with blood vessels, immune cells, and cytokines in the TME, contributing to drug resistance (Gao et al., 2023). Furthermore, BCSCs possess self-renewal and multilineage differentiation capabilities, which may lead to recurrence and metastasis after treatment (Zhou et al., 2021). Genetic mutations and epigenetic alterations, such as DNA methylation and histone modifications, are closely linked to the characteristics of BCSCs (Tian et al., 2021). Hence, developing therapeutic strategies targeting BCSCs is crucial for improving the prognosis of breast cancer (Baumann et al., 2008; Zhang et al., 2017; Zhang L. et al., 2023).

In the field of oncology, chemotherapy is a cornerstone of treatment, but the application of synthetic small molecule drugs is not without significant drawbacks. Doxorubicin, a widely used drug for breast cancer treatment, can lead to serious adverse reactions such as cardiotoxicity and myelosuppression with long-term use. Additionally, it can develop resistance through mechanisms such as the activation of the non-canonical NF-kB signaling pathway (Chong et al., 2021; Lim et al., 2023). Therefore, the scientific community has begun to shift its research focus towards naturally derived and edible small molecule compounds, such as flavonoids. These substances typically exhibit non-toxicity or non-mutagenicity to normal cells. Flavonoids can inhibit the proliferation of tumor cells while having minimal impact on normal cells, and they exhibit non-mutagenicity in standard mutagenicity tests such as the Ames test. Furthermore, they possess excellent antioxidant activity and immune-modulating effects, making them strong candidates for the development of novel anti-cancer therapies (Yan et al., 2017). Apigenin (API), widely present in plants, belongs to a subclass of flavonoids (Salehi et al., 2019). Research extensively documents the potent anti-inflammatory and antitumor properties of API (Funakoshi-Tago et al., 2011; Madunić et al., 2018; Tian et al., 2021; Ji et al., 2023), which has led to its application in the treatment of various cancers, including breast cancer. The API has been demonstrated to substantially decrease yes-associated protein - transcriptional co-activator with PDZ-binding motif (YAP/TAZ) activity and downregulate the expression of target genes, including connective tissue growth factor (CTGF) and cysteine-rich angiogenic inducer 61 (CYR61), in triple negative breast cancer (TNBC) stem cells (Li et al., 2018). In addition, it has been suggested that the API can inhibit the dedifferentiation properties of TNBC cells by inhibiting sirtuin 3 (SIRT3) and sirtuin 6 (SIRT6) proteins (Sharma et al., 2022). However, the effect of API on the BCSCs of estrogen receptor-positive breast cancer has not been reported. Therefore, in this project, we focused on the effect and mechanism of the API against estrogen-positive BCSCs, which is essential for the effective treatment of breast cancer. The Michigan cancer foundation-7 (MCF-7) cell line is derived from breast cancer and is hormone receptor-positive, specifically characterized by estrogen receptor (ER) and progesterone receptor (PR) positivity. MCF-7 cells are a human breast cancer adenocarcinoma epithelial cell line widely used in breast cancer research (Theodossiou et al., 2019).

In order to bridge this gap in knowledge, our study undertook a comprehensive investigation and validation of the effects and underlying molecular mechanisms of API against BCSCs derived from MCF-7 cells, employing an integrated approach that combined network pharmacology, molecular docking, as well as a series of *in vitro* experiments. The objective of our study was to determine the potential therapeutic effects of API on BCSCs and to uncover the molecular mechanisms underlying its activity against these cells. Network pharmacology facilitated a comprehensive understanding of the complex interactions between API and its molecular targets, including the intricate pathways and networks involved in its anti-cancer effects. Molecular docking allowed us to examine the molecular interactions between API and its putative targets at the atomic level, yielding critical insights into the binding mechanisms that govern drug-target interactions. Furthermore, our comprehensive experimental approach permitted the verification of computational predictions and further dissected the cellular and molecular processes affected by API treatment. Empirical evidence supporting the anti-BCSC effects of API was furnished by these experiments, demonstrating its ability to modulate key signaling pathways, induce apoptosis, and inhibit proliferation and self-renewal of these stem-like cells.

## 2 Materials and methods

### 2.1 Drugs and reagents

The cell lines MCF-7 and human umbilical vein endothelial cells (HUVECs) were sourced from the Shanghai Cell Bank, Chinese Academy of Sciences (Shanghai, China). API (98%) was purchased from Shanghai Macklin Biochemical Co., LTD. (Shanghai, China). Salinomycin (SAL) (98%) was purchased from MedChemExpress (Monmouth Junction, NJ, United States). A 0.05% trypsin solution, containing ethylene diamine tetraacetic acid (EDTA) dissolved in PBS, was procured from Wuhanpssel Life Technology Co., LTD. (Wuhan, China). Dulbecco’s modified eagle medium (DMEM), fetal bovine serum (FBS), penicillin-streptomycin solution, and goat serum were purchased from Gibco Life Technologies (Grand Island, NE, United States). Endothelial cell complete medium was sourced from VivaCell Biotechnology GmbH (Denzlingen, Germany). Epidermal growth factor (EGF) and basic fibroblast growth factor (bFGF) were purchased from PeproTech (Hamburg, Germany). Heparin was sourced from Sigma-Aldrich Chemical Company (St. Louis, MO, United States). The B27 supplement was acquired from Gibco (Grand Island, NY, United States). A cell counting kit-8 (CCK-8) was purchased from Apexbio (Houston, TX, United States). An Annexin V-FITC/PI apoptosis kit was procured from Beyotime Biotechnology (Shanghai, China). The ALDEFLUORTM kit for aldehyde dehydrogenase 1 family member (ALDH1) activity assays was purchased from STEMCELL Technologies China Co., LTD. (Shanghai, China). The PE-conjugated mouse anti-human cluster of CD24 molecule (CD24) antibody (Clone ML5) and the FITC-conjugated mouse anti-humancluster of CD44 molecule (CD44) antibody (Clone G44-26) were purchased from BD Biosciences (San Diego, CA, United States).

### 2.2 Collection of gene targets for API and BCSC-related targets

Using “Apigenin” as the keyword, we searched for gene targets associated with API across multiple databases, including traditional chinese medicine systems pharmacology database and analysis platform (TCMSP) (https://tcmsp-e.com) (Ru et al., 2014), the encyclopedia of traditional chinese medicine (ETCM) (http://www.tcmip.cn/ETCM/index.Php) (Xu et al., 2019), Pharm Mapper (http://www.lilab-ecust.cn/pharmmapper) (Wang et al., 2017), SwissTarget Prediction (https://swisstargetprediction.ch) (Daina et al., 2019) and GeneCards (https://www.genecards.org) (Stelzer et al., 2016). In addition, BCSC-related targets were collected from the GeneCards database (https://www.genecards.org) (Stelzer et al., 2016), Cancer SEA (hrbmu.edu.cn) (Yuan et al., 2019) and the NCBI Count database (https://www.ncbi.nlm.nih.gov/gene) (Sharma et al., 2018), specify the species-specific characteristics of “*Homo sapiens*” in the search parameters. To identify possible common targets of API and BCSCs, the two targets were crossed.

### 2.3 Construction of PPIs network

The predicted potential common targets were imported into the STRING database (https://www.string-db.org/) (Szklarczyk et al., 2023) for protein-protein interactions (PPIs) analysis. To validate the veracity and precision of our data analysis, we established a stringent criterion by setting the minimum interaction score to a high confidence level of 0.9 and the species was restricted to “*Homo sapiens*”. Subsequently, the results were integrated into Cytoscape 3.9 for visualization of the interaction network between common protein targets, with nodes having a connectivity score of 0 being omitted. The cytoNCA plugin facilitated the calculation of degree values across various pathways, while the cytoHubba plugin employed a topological network algorithm to assign values to each gene. Our examination focused on the top 10 targets with the highest maximum clique centrality (MCC), aiming to discern specific pathways and pivotal targets that may be significantly influenced by API in the context of BCSCs. This approach was essential for uncovering the potential impact of API on BCSC biology and identifying key molecular intervention points.

### 2.4 GO and KEGG enrichment analysis

The DAVID database was utilized for conducting both a gene ontology (GO) functional enrichment analysis and a Kyoto encyclopedia of genes and genomes (KEGG) signaling pathway enrichment analysis for the common targets of API and BCSCs (Sherman et al., 2022). The GO functional enrichment analysis encompassed three primary ontologies: molecular function (MF), biological process (BP), and cellular component (CC). Filter the results with *p*-values less than 0.01. Select the top 10 GO terms and 20 KEGG pathways, and then use SRplot (http://www.bioinformatics.com/) (Tang et al., 2023) for graphical representation. SRplot is a tool for data visualization and graphical depiction.

### 2.5 Construction of component-target-pathway network

We integrated potential API targets, BCSC-associated targets, and enriched pathways into Cytoscape 3.9 to construct the “component-target-pathway” network. This network uses distinct graphical nodes and color-coding to visually represent its various components, targets, and pathways, whereas the interaction between any two nodes is denoted by connected edges. This approach facilitated a comprehensive visualization of the interactions and relationships within the network, providing a deeper understanding of the complex biological processes at play.

### 2.6 Molecular docking

Molecular docking between key targets and API was conducted utilizing AutoDock software (https://vina.scripps.edu/). The binding affinity value, expressed in kcal/mol, corresponds to the thermodynamic binding affinity of API for the target protein. An increased absolute value of the binding free energy indicates greater stability of the ligand-receptor complex. Having identified the precise location of the original ligand of the receptor protein, we proceeded to remove it. Following this, we defined the dimensions of the grid box to 30 in the x, y, and z dimensions, with a modes parameter of 20, an exhaustiveness parameter of 15, and a number of genetic algorithm runs (GA) set to 10. We then performed molecular docking of the original ligand and the API with the receptor protein. By comparing the binding energies of the API and the original ligand, we assessed the binding stability of the API with the key target, which provided valuable insights into the potential efficacy of the API as a therapeutic agent.

### 2.7 Cell culture

MCF-7 cells were cultured in DMEM supplemented with 10% FBS and 1% penicillin-streptomycin solution. HUVECs were maintained in endothelial cell complete medium. All cultures were incubated at 37°C in a humidified atmosphere containing 5% CO_2_. BCSCs were derived from MCF-7 cells using a suspension culture method, as previously reported (Cao et al., 2018). MCF-7 cells were cultured in 6-well ultra-low adherent plates (Corning, NY, United States) at a density of 2 × 10^5^ cells per well. Each well contained 2 mL of DMEM supplemented with 2% B27, 1% penicillin-streptomycin solution, 0.4% bovine serum albumin (BSA), 20 ng/mL EGF, bFGF, and 10 ng/mL heparin, and was maintained at 37°C with 5% CO_2_ in air. Following an uninterrupted culture period of at least 3 weeks, stem cells were successfully cultured and assessed for stmness indices.

### 2.8 Cell proliferation assay

The CCK-8 assay was utilized to assess the cytotoxic effects of API and SAL on enriched BCSCs and MCF-7 microspheres. Enriched BCSCs and MCF-7 microspheres were dissociated into single-cell suspensions and then seeded into 96-well plates at a density of 5 × 10^3^ cells per well. Various concentrations of API were added to each well, with the BCSC experimental group exposed to concentrations ranging from 5 to 80 μM, and the MCF-7 group to concentrations ranging from 5 to 100 µM. Concurrently, a blank control group was included and all plates were incubated for 48 h. Similarly, SAL was added to each well at concentrations ranging from 0 to 10 µM and incubated under identical conditions. Following the incubation period, 10 µL of CCK-8 reagent was added to each well, and the cells were further incubated at 37°C for 3 h. The optical density was measured at 450 nm using a Synergy H1 Hybrid multimode microplate reader. The half-maximal inhibitory concentration (IC_50_) values of API and SAL on BCSCs and MCF-7 cells were subsequently calculated using GraphPad Prism 9.5 software.

To ascertain whether API exhibits greater selectivity for BCSCs relative to normal cells, HUVECs were prepared for assessment of their response to API. The HUVECs were allocated into groups: a control group with no treatment, an API treatment group with concentrations ranging from 5 to 100 μM, and a SAL treatment group with concentrations ranging from 0.25 to 20 µM. Following the same experimental protocol, the IC_50_ values for API and SAL on HUVECs were calculated. This analysis aimed to evaluate the selectivity of API for BCSCs over normal cells.

### 2.9 Experiments to inhibit sphere formation and induce apoptosis

A 24-well ultra-low adhesion plate was filled with 3 mL of serum-free medium DMEM/F12^+^. BCSCs derived from MCF-7 cells were seeded into the wells at a density of 5 × 10^4^ cells/well and subjected to drug treatment. The positive control group received a single concentration equivalent to the IC_50_ value, which is 2 µM of SAL. In contrast, the experimental groups were designed at different concentration gradients based on the IC_50_ value, specifically at 1/4, 1/2, and 1 times the IC_50_ value, corresponding to 7 μM, 14 μM, and 28 µM of the API, respectively. Additionally, a negative control (NC) group was included to ensure the accuracy and reliability of the experimental results. Each condition was assayed in triplicate, and the cells were incubated for 6 days without medium replacement. Microscopic images were captured on days 0, 3 and 6 to monitor the effects of the treatments on spheroid formation. Only spheres with a cell mass/spherical area greater than or equal to 5,000 µM^2^ were quantified.

BCSCs treated with the aforementioned drugs were collected, and 6 × 10^5^ cells were resuspended in 200 µL of binding buffer. Annexin V-FITC and PI staining solutions (5 µL each) were added, and the cells were incubated for 15 min in the dark at room temperature. Subsequently, the stained cells were subsequently analyzed using a CytofLEX S flow cytometer, employing the following gating strategy: First, cells were gated using forward scatter (FSC) and side scatter (SSC) to separate the cellular population from debris. Then, single cells were selected by gating on FSC-A (area) vs. FSC-H (height) to exclude doublets and aggregates. Lastly, apoptotic cells were identified using an FITC (Annexin V-FITC) vs. PI plot, categorizing live cells as Annexin V−/PI−, early apoptotic as Annexin V+/PI−, late apoptotic or necrotic as Annexin V+/PI+, and dead cells as Annexin V−/PI+. The percentage of apoptotic cells was calculated for each group using CytExpert software after Annexin V-FITC/PI staining.

### 2.10 Flow cytometric assessment of BCSCs markers

To determine the frequency of CD44^+^/CD24^−/low^ cells in MCF-7 cells and BCSCs, we conducted a flow cytometric analysis. Cells were harvested and stained with phycoerythrin (PE)-conjugated CD24 and fluorescein isothiocyanate (FITC)-conjugated CD44 antibodies following a standard protocol. We selected 2.5 × 10^5^ cells per sample, washed them twice with 1 mL staining buffer, and centrifuged at 250 × g for 3 min. After supernatant removal, the cell pellet was resuspended in 100 µL staining buffer containing 2.5 µL each of PE-conjugated CD24 and FITC-conjugated CD44 antibodies. After a 30-min incubation at 4°C, the stained cells were then analyzed using a CytofLEX S flow cytometer with the following gating strategy: Initially, cells were gated on FSC and SSC to distinguish the cellular population from debris. Next, single cells were selected by gating on FSC-A vs. FSC-H to exclude doublets and aggregates. Finally, CD44^+^/CD24^−/low^ cells were identified using an FITC (CD44-FITC) vs. PE (CD24-PE) plot.

To quantify the ALDH^high^ population in MCF-7 cells and BCSCs, we utilized the ALDEFLUOR™ kit for staining. Harvested cells (6 × 10^5^) were resuspended in 600 µL of ALDEFLUOR™ assay buffer, and 3 µL of activated ALDEFLUOR™ reagent was added. A control group was prepared by mixing half of the cell suspension with 6 µL of the ALDH inhibitor, diethylaminobenzaldehyde (DEAB). Following a 45-min incubation at 37°C, cells were centrifuged at 250 × g for 3 min, and the supernatant was aspirated. Cell pellets were resuspended in 300 µL of ALDEFLUOR™ assay buffer, and ALDH activity was measured using the CytofLEX S flow cytometer. The stained cells were analyzed with the following gating strategy: Initially, cells were gated on FSC and SSC to distinguish the cellular population from debris. Next, single cells were selected by gating on FSC-A vs. FSC-H to exclude doublets and aggregates. Finally, the ALDH^high^ population was identified using an FL-1 (green fluorescence) vs. SSC plot.

### 2.11 Q-PCR

First, total RNA was extracted from cells that had been treated with various drug concentrations for 48 h. RNA purity and quality were assessed to ensure suitability for downstream applications. Subsequently, reverse transcription and amplification of quantitative polymerase chain reaction (Q-PCR) were conducted using the established reaction system. We investigated whether the API could downregulate the expression of genes associated with stemness at the RNA level, including ALDHA1A1, MYC proto-oncogene, bHLH transcription factor (c-MYC), nanog homeobox (NANOG), and epithelial cell adhesion molecule (EpCAM), and designed corresponding primers based on the top 10 targets selected by MCC, thereby laying the groundwork for further investigation into the mechanism of API against BCSCs. Glyceraldehyde 3-phosphate dehydrogenase (GAPDH) was utilized as an endogenous control for data normalization. Data were analyzed using the 2^−ΔΔCT^ method, enabling precise quantification of gene expression levels. The primer sequences, designed specifically for this study, are detailed in Table 1. This approach ensures the accuracy and reliability of the gene expression analysis.

**TABLE 1:** Primer sequences of Q-PCR.

Gene	Sense primer (5′-3′)	Antisense primer (5′-3′)
GAPDH	ACA​GCC​TCA​AGA​TCA​TCA​GCA​AT	CTT​CTG​GGT​GGC​AGT​GAT​GG
ALDH1A1	TAG​CTG​ATG​CCG​ACT​TGG​AC	TCT​TAG​CCC​GCT​CAA​CAC​TC
EPCAM	GCG​AGT​GAG​AAC​CTA​CTG​GA	CGC​GTT​GTG​ATC​TCC​TTC​TG
NANOG	AAT​GGT​GTG​ACG​CAG​AAG​GC	GTG​CAC​CAG​GTC​TGA​GTG​TT
CD44	TCA​ATG​CTT​CAG​CTC​CAC​CT	GGT​GCC​ATC​ACG​GTT​AAC​AA
SOX2	AAC​CAG​CGC​ATG​GAC​AGT​TA	CGA​GCT​GGT​CAT​GGA​GTT​GT
c-MYC	AGT​GGA​AAA​CCA​GCA​GCC​TC	TTC​TCC​TCC​TCG​TCG​CAG​TA
TP53	AAA​CAA​CGT​TCT​GTC​CCC​CTT	GGG​AGC​TTC​ATC​TGG​ACC​TG
HSP90AA1	GTG​TCA​GTC​ACC​AAA​GAA​GGC	CGG​TTT​GAC​ACA​ACC​ACC​TTT
HSP90AB1	TTT​ATT​CCT​CGT​CGG​GCT​CC	ACC​ACA​CCA​CGG​ATA​AAA​TTG
HIF1A	AGA​TTT​TGG​CAG​CAA​CGA​CAC	CGT​TTC​AGC​GGT​GGG​TAA​TG
ESR1	GAA​GAG​GGT​GCC​AGG​CTT​T	CGC​CAG​ACG​AGA​CCA​ATC​AT
AKT	GCT​GCA​CAA​ACG​AGG​GGA​G	CCT​CAC​GTT​GGT​CCA​CAT​CC
EGFR	GAG​AAC​TGC​CAG​AAA​CTG​ACC	GTG​GCT​TCG​TCT​CGG​AAT​TTG
BCL2	CCG​CGA​CTC​CTG​ATT​CAT​TG	AGT​CTA​CTT​CCT​CTG​TGA​TGT​TG
GSK3B	TCC​AGT​GGT​GAG​AAG​AAA​GAT​GA	GCG​TCT​GTT​TGG​CTC​GAC​TA
TERT	ATG​TCA​CGG​AGA​CCA​CGT​TT	ACC​CTC​TTC​AAG​TGC​TGT​CTG​A

### 2.12 Western blot analysis

By integrating Q-PCR expression data with KEGG signaling pathway enrichment analysis, we identified key signaling pathways. Specifically, we focused on validating the expression and activity of key proteins phosphatidylinositol-4,5-bisphosphate 3-kinase (PI3K), AKT serine/threonine kinase (AKT), and tumor protein 53 (p53) at the protein level. For the assessment of cellular stemness, we used GAPDH as an internal control to evaluate in depth the expression of stem cell markers at the protein level, including ALDH1A1, c-MYC, NANOG, and EpCAM. Furthermore, to elucidate the mechanism of action of the API, we detected the expression levels of phosphorylated PI3K (p-PI3K), phosphorylated AKT (p-AKT), and p53 proteins.

Cells were exposed to varying concentrations of the API and the positive control, SAL (2 µM), for a duration of 48 h. Following this incubation period, the residual medium was carefully aspirated, and the cells were gently rinsed. Cells were then lysed using a radioimmunoprecipitation assay (RIPA) buffer supplemented with a protease inhibitor cocktail (phenylmethylsulfonyl fluoride, PMSF) at a ratio of 100:1 for 20 min at 4°C. The lysate was then centrifuged at 12,000 g and 4°C for 10 min, and the supernatant was collected. The protein concentration in the supernatant was then determined using the bicinchoninic acid (BCA) assay, which involved preparing a standard curve by diluting a 0.5 mg/mL protein standard to concentrations of 0.5, 0.25, 0.125, and 0.0625 mg/mL and adding 10 µL of each to a 96-well plate. Following this, the protein samples were diluted tenfold by mixing 1 µL of sample with 9 µL of water and added to the plate. Then, 100 µL of BCA reagent was added to each well, incubated at 37°C for 30 min, after which the absorbance was measured at 562 nm. The standard curve was plotted using the absorbance values, and the protein concentrations of the samples were interpolated from it.

For the Western blot analysis, proteins were resolved on a 10% sodium dodecyl sulfate-polyacrylamide (SDS-PAGE) gel. The amount of protein was 30 mg per well. The resolved proteins were transferred onto polyvinylidene fluoride (PVDF) membranes, followed by blocking with a 5% solution of skim milk in tris-buffered SALine with Tween (TBST) for 1 h at ambient temperature. The membranes were incubated with the following primary antibodies overnight at 4°C, diluted as specified: anti-ALDH1A1 (1:500), anti-c-MYC (1:1000), anti-NANOG (1:1000), anti-EpCAM (1:1000), anti-phospho-PI3K (1:1000), anti-PI3K (1:1000), anti-phospho-AKT (1:1000), anti-AKT (1:1000), anti-tumor protein p53 (TP53) (1:1000), and anti-GAPDH (1:3000). After thorough washing with TBST buffer, the membranes were incubated with a secondary horseradish peroxidase (HRP)-conjugated goat anti-rabbit IgG antibody (1:2000) for 1 h at room temperature. After additional washes with TBST, the membranes were visualized using an ultra-sensitive enhanced chemiluminescence (ECL) detection kit reagent and imaged using a MiniChemi™ 910 imaging system. The intensity of the bands was quantified using VisionWorks software (UVP, Upland, CA) to assess the relative protein expression levels.

### 2.13 Statistical analysis

In this study, the data were derived from at least 3 distinct experimental trials, ensuring a robust dataset for analysis. Prior to statistical analysis, the data were subjected to rigorous examination to assess their distribution and variance. To standardize the data and reduce variability, logarithmic transformations were applied to all variables. Statistical analysis involved one-way analysis of variance (one-ANOVA) to evaluate differences among multiple groups, and *post hoc* pairwise comparisons were conducted using Tukey’s honestly significant difference (HSD) test to precisely identify which specific groups differed significantly from one another. Results are expressed as mean ± standard deviation (Mean ± SD), which not only clearly delineates the central tendency of the data points but also reflects their dispersion. A *p*-value less than 0.05 was considered statistically significant.

## 3 Results

### 3.1 Unveiling the therapeutic potential of API on BCSCs through network pharmacology analysis

**FIGURE 1:**
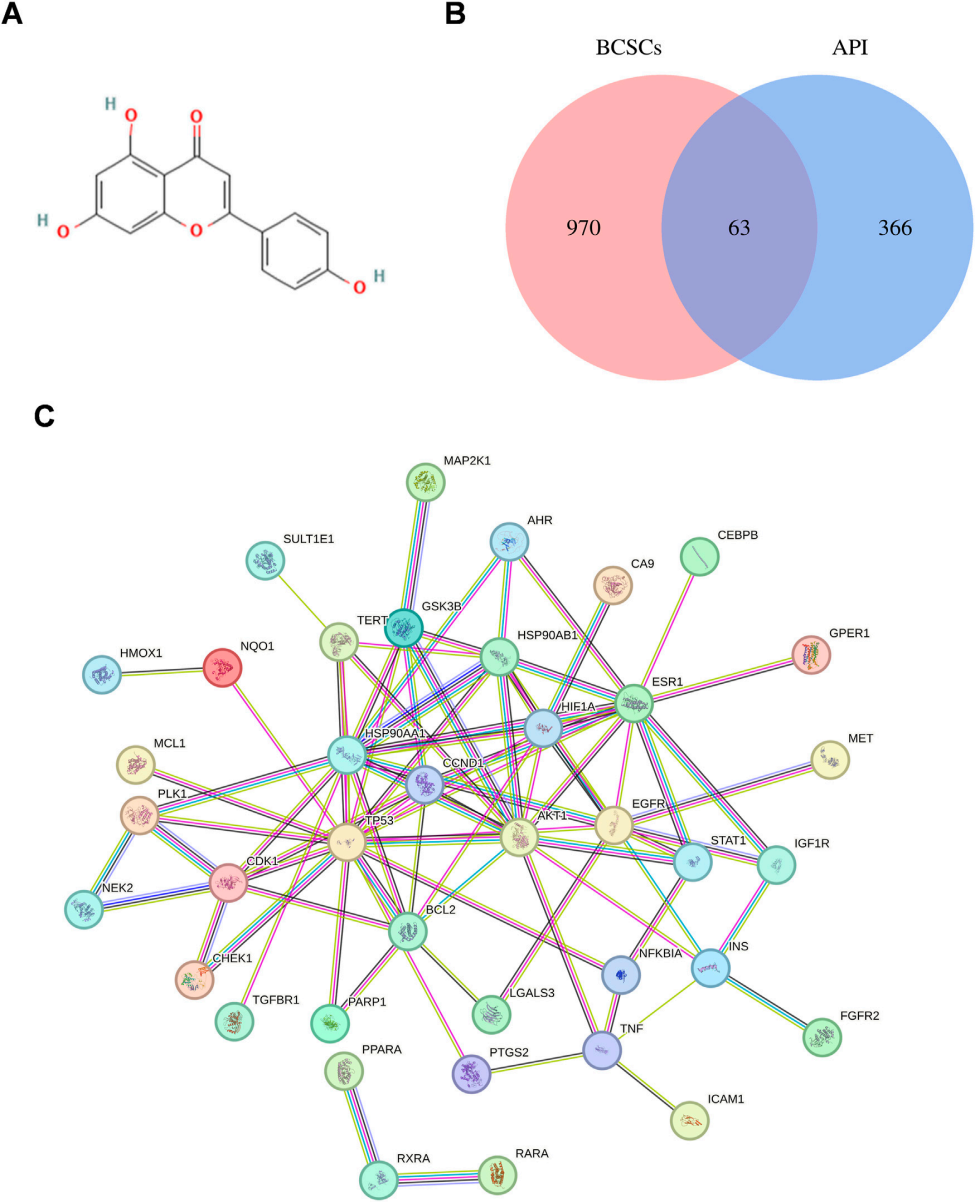
Exploration of API’s Impact on BCSCs via network pharmacology. **(A)** Chemical structure of API. **(B)** Venn diagram of API and BCSCs target overlap. **(C)** Protein-protein interaction network constructed with STRING Database, illustrating the connectivity and interaction patterns among the overlapping targets between API and BCSCs.

**FIGURE 2:**
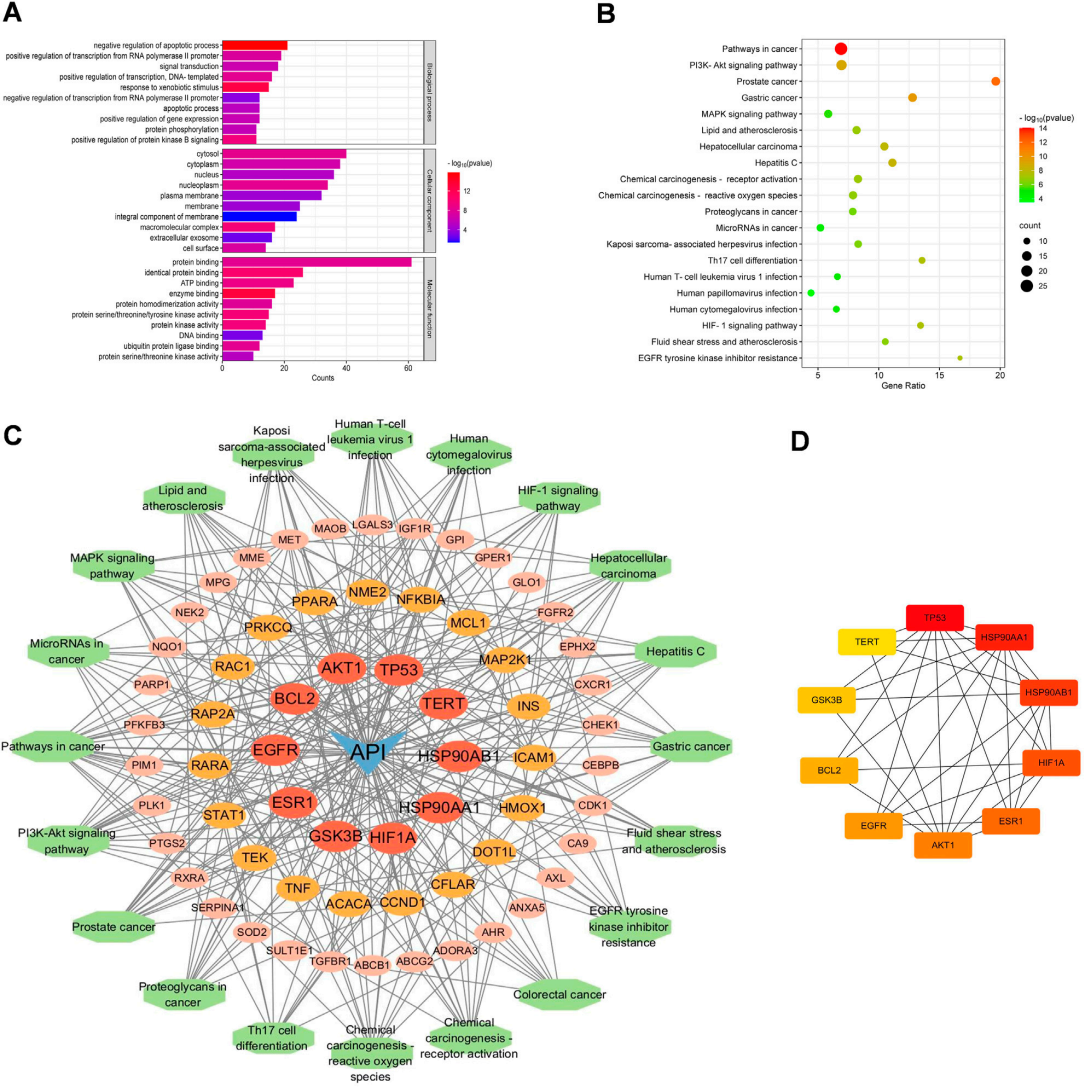
Functional enrichment analysis of core targets for API against BCSCs. **(A)** The top 10 GO enrichment terms of core targets for API against BCSCs (BP: biological process, CC: cellular components, MF: molecular function). **(B)** Top 20 KEGG enrichment terms of core targets for API against BCSCs. **(C)** “Component-target-pathway” network diagram, node size is positively correlated with node degree value. **(D)** Top 10 core targets ranked by maximum clique centrality (MCC) score in the PPI network.

To elucidate the potential effects of API against BCSCs, a comprehensive network pharmacology study was conducted utilizing established and authoritative databases. Initial confirmation of API’s structure (Figure 1A) was followed by extraction of two datasets: 1033 BCSC-associated target genes and 429 API-associated target genes from databases such as Genecards. Through the construction of a venn diagram, an overlapping set of 63 genes was identified (Figure 1B). For PPIs, the STRING database and Cytoscape 3.9 software were employed. Employing the cytoHubba plugin, the topological significance of the 63 potential core targets was identified and ranked to reveal hub genes and subnetworks (Figure 1C). Subsequent GO and KEGG enrichment analysis revealed significant enrichment of these targets in 117 pathways, with the PI3K/AKT pathway being the obvious enriched (Figures 2A, B). A “compound-target-pathway” network was generated using Cytoscape 3.9, integrating the potential API targets, BCSC targets, and enriched pathways. Within this network, the size and color of the nodes represent their level of importance (Figure 2C). Notably, TP53, heat shock protein 90 alpha family class A member 1(HSP90AA1), heat shock protein 90 alpha family class B member 1 (HSP90AB1) hypoxia inducible factor 1 subunit alpha (HIF1A), estrogen receptor 1 (ESR1), AKT1, epidermal growth factor receptor (EGFR), BCL2 apoptosis regulator (BCL2), glycogen synthase kinase 3 beta (GSK3B), and telomerase reverse transcriptase (TERT) emerged as prominent, occupying the top 10 target positions (Figure 2D; Table 2). The central role of TP53 as the most critical nuclear target and its high enrichment strongly indicates that API may play a crucial role in combating BCSCs via the “PI3K/AKT/p53” signaling pathway.

**TABLE 2:** Information of top 10 core targets.

Target	Name	Score	Degree	Betweenness	Closeness
TP53	Tumor protein p53	356	9.0	7.23	1.00
HSP90AA1	Heat shock protein 90 alpha family class A member 1	331	9.0	7.23	1.00
HSP90AB1	Heat shock protein 90 alpha family class B member 1	294	8.0	4.23	0.90
AKT1	AKT serine/threonine kinase	214	8.0	4.83	0.90
HIF1A	Hypoxia inducible factor 1 subunit alpha	265	7.0	2.07	0.82
ESR1	Estrogen receptor 1	260	6.0	0.40	0.75
EGFR	Epidermal growth factor receptor	128	5.0	0.00	0.69
TERT	Telomerase reverse transcriptase	25	4.0	0.00	0.64
BCL2	BCL2 apoptosis regulator	45	4.0	0.00	0.64
GSK3B	Glycogen synthase kinase 3 beta	31	4.0	0.00	0.64

### 3.2 Molecular docking identifies interactions of top 10 proteins targets and API

**FIGURE 3:**
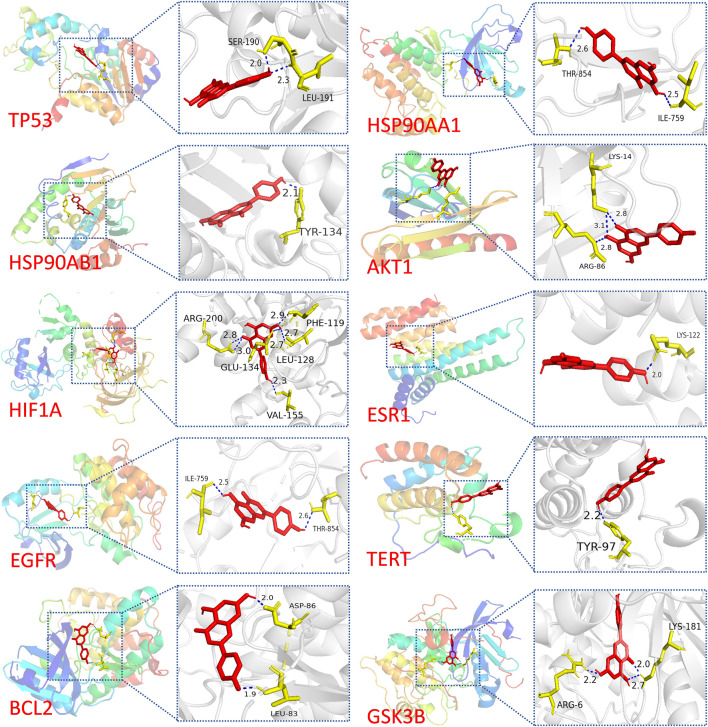
Visualization of molecular docking results. Molecular docking of TP53, HSP90AA1, HSP90AB1, AKT1, HIF1A, ESR1, EGFR, TERT, BCL2, and GSK3B with API.

Molecular docking analysis was conducted to explore the potential targets of API against BCSCs. Based on network pharmacology findings, API was docked with the top 10 proteins, ranked by MCC scores, and the binding energy was calculated. A lower binding energy signifies a more stable ligand-receptor configuration, as reflected in the docking score. The binding energy of API was compared with that of the original ligands for each core target to evaluate stable binding. A binding energy close to or lower than −5 kcal/mol (Nimbannavar and Mane, 2019; Li et al., 2023; Ray and Mukherjee, 2024), suggests stable binding between API and the target protein. The results indicated that 55% of the targets had a docking energy below −8 kcal/mol, 27% below −7 kcal/mol, 9% below −6 kcal/mol, and another 9% below −5 kcal/mol. All binding energies were below −5 kcal/mol, demonstrating potential favorable binding activity of the top 10 targets with API (Table 3). The cartoon representation enhances visualization of the ligand-protein binding residues and illustrates the molecule’s propensity to form hydrogen bonds (Figure 3).

**TABLE 3:** The binding energies of API or co-crystallized ligands with related protein targets.

Name	PDB ID	Binding energy (kcal/mol)	
		API	Co-crystallized ligands
TP53	1YC5	−8.6	−5.6
HSP90AA1	2YK9	−8.9	−13.1
HSP90AB1	6N8Y	−8.9	−8.1
AKT1	1UNQ	−5.2	−7.1
HIF1A	6GMR	−7.0	−5.7
ESR1	7B9R	−6.5	−6.7
EGFR	8A27	−8.4	−9.5
TERT	4QB3	−7.2	−7.6
BCL2	5MHQ	−7.2	−8.5
GSK3B	1O6L	−9.1	−10.0

Notably, a significant difference in binding energy was observed between the original ligand of TP53 and API compared to other targets. Specifically, the binding energy of API to TP53 exhibited a more pronounced decrease compared to the original ligand, indicating a theoretical indication of tighter binding to TP53. This observation implies that APT might have potential effect with TP53.

### 3.3 MCF-7 cells exhibit enhanced stemness following suspension culture

**FIGURE 4:**
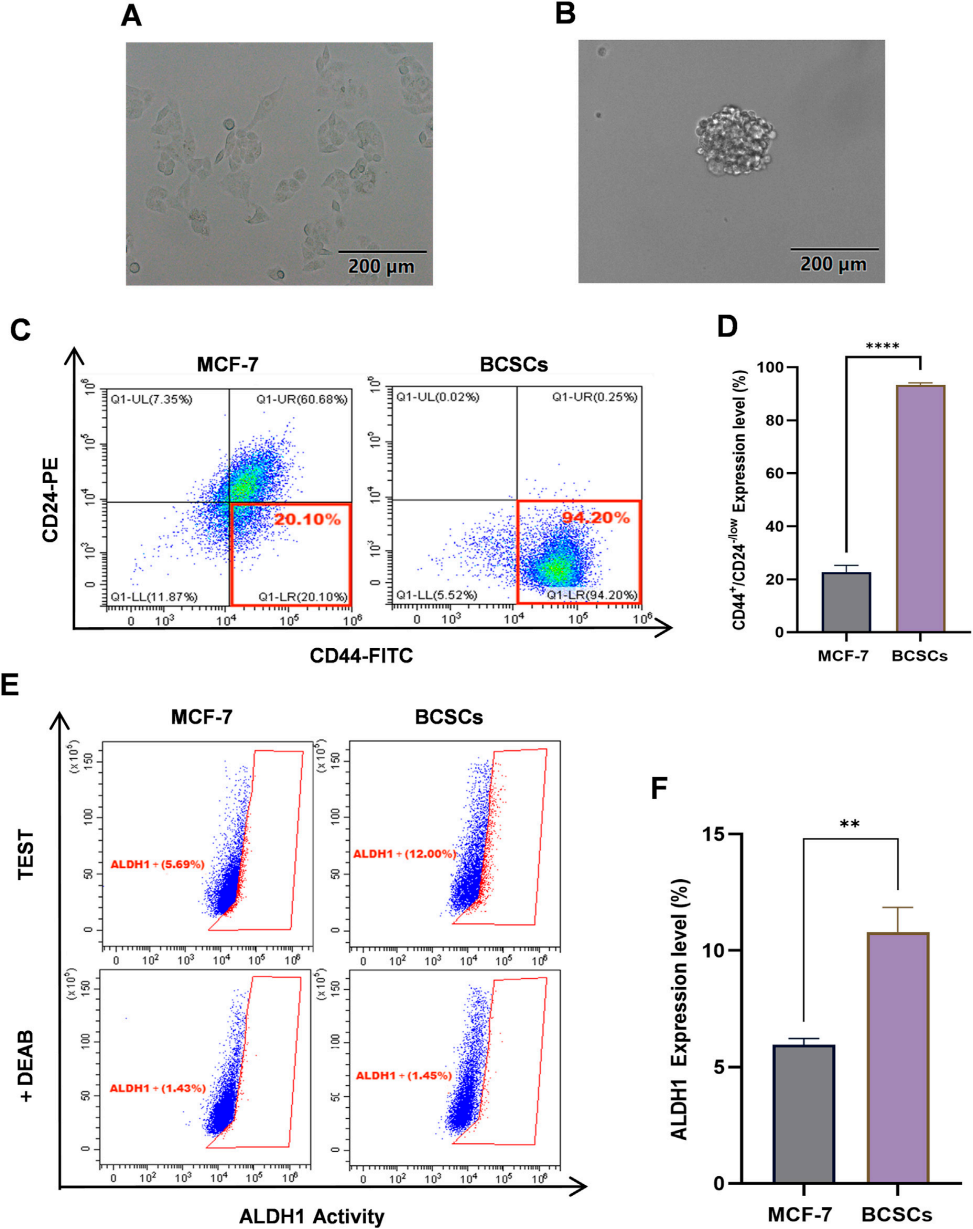
BCSCs derived from MCF-7 cells by suspension culturing. **(A)** The morphology of the MCF-7 cells. **(B)** The morphology of BCSCs derived from MCF-7 cells by suspension culturing. **(C, D)** The percentage of CD44^+^/CD24^−/low^ subset in MCF-7 cells and BCSCs-derived from MCF-7 cells by suspension culturing. **(E, F)** The ALDH1 expression level of MCF-7 cells and BCSCs-derived from MCF-7 cells by suspension culturing. “***”, *p <* 0.001; “****”, *p <* 0.0001, α = 0.05.

MCF-7 cells cultured under serum-free conditions in DMEM/F12^+^ medium were subjected to a 3-week suspension culture in ultra-low attachment plates. Over the course of this period, cells progressively aggregated to form tumorospheres. Tumorosphere formation was observed and documented using microscopy (Figures 4A, B). To evaluate stemness properties, the proportions of CD44^+^/CD24^−/low^ and ALDH1^high^ subsets were determined by flow cytometry. Results demonstrated that following suspension culture in serum-free medium DMEM/F12^+^, the percentage of CD44^+^/CD24^−/low^ significantly increased from 22.76% ± 2.52% to 93.38% ± 0.65%, which was significantly higher compared to MCF-7 cells (*p <* 0.0001, 95% CI: [67.80, 73.35]) (Figures 4C, D). Furthermore, the expression level of ALDH1 rose from 5.69% ± 0.20% to 12.00% ± 1.00%, which is also a significant increase (*p <* 0.01, 95% CI: [3.05, 6.70]) (Figures 4E, F). Collectively, these findings suggest that BCSCs derived from MCF-7 cells through suspension culture exhibit robust stem-like characteristics.

### 3.4 API effective suppresses the proliferation BCSCs and induced their apoptosis

**FIGURE 5:**
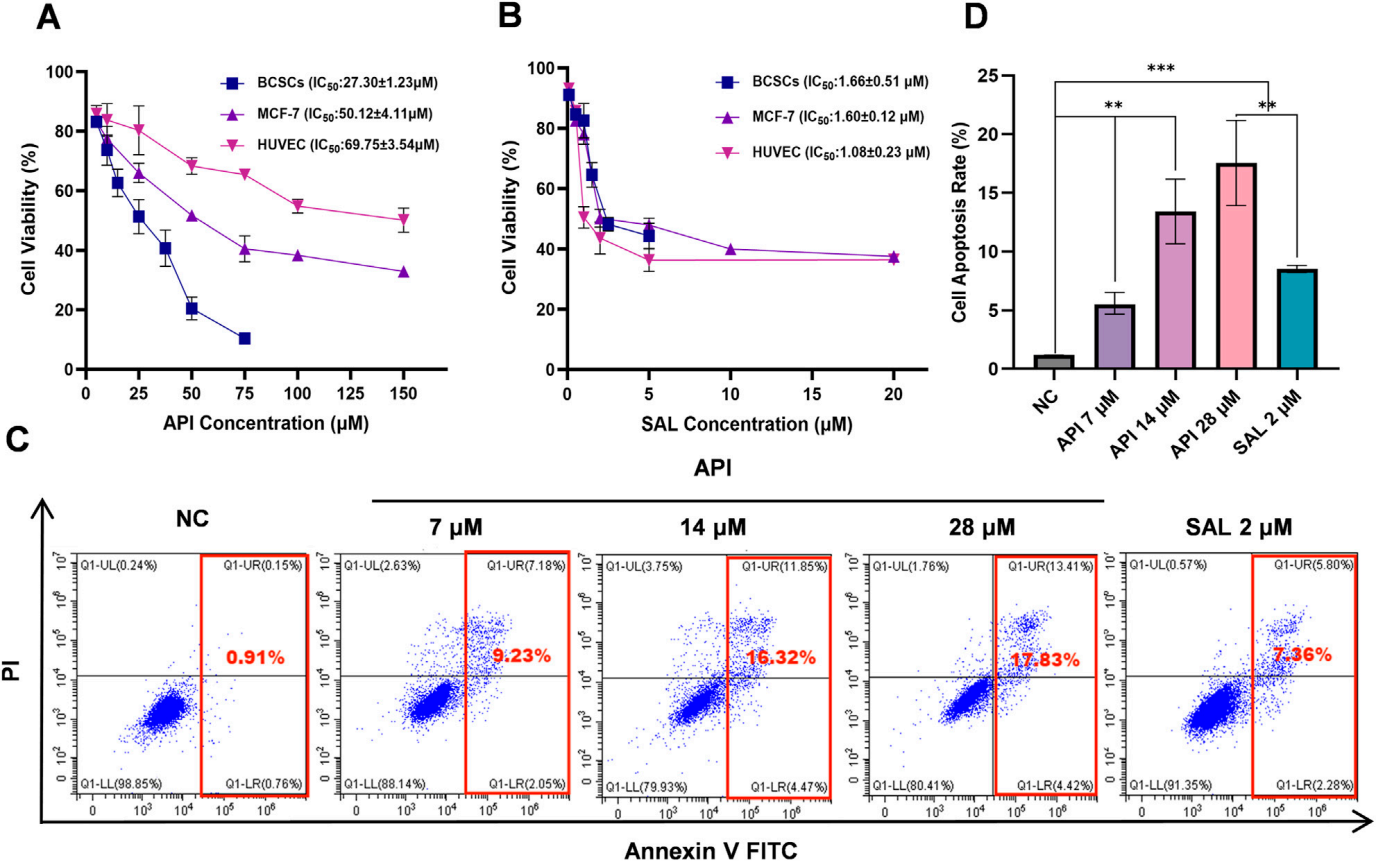
API effectively suppresses the proliferation and induces apoptosis of BCSCs. **(A, B)** The inhibitory effects of API **(A)** and SAL **(B)** on the proliferation of BCSCs, MCF-7, and HUVEC. **(C, D)** The apoptotic effects of API and SAL on BCSCs. “**”, *p <* 0.01; “***”, *p <* 0.001, α = 0.05.

To elucidate the effect of API on the proliferation of BCSCs more clearly, we tested various concentrations of API on these cells and conducted comparative experiments using HUVECs to evaluate the specificity of API’s cytotoxicity. After 48 h of treatment, we observed a significant dose-dependent inhibition of BCSCs’ proliferation. Specifically, as the concentration of API increased from 0 µM to 100 μM, the cell viability of BCSCs, MCF-7, and HUVEC all significantly decreased. Through CCK-8 assays, we calculated the IC_50_ values of API in BCSCs, MCF-7, and HUVECs to be 27.30 ± 1.23 µM, 50.12 ± 4.11 µM, and 69.75 ± 3.54 µM, respectively. The IC_50_ value of API for BCSCs was approximately 2.55 times lower than that for HUVECs and 1.84 times lower than that for MCF-7 cells, indicating a significant selective cytotoxicity of API for BCSCs. Furthermore, while stem cells are generally more drug-tolerant, in this study, BCSCs exhibited greater sensitivity to API compared to non-stem breast cancer cells. For instance, under the influence of 50 µM API, the average viability of MCF-7 cells was 56.78%, while the average viability of BCSCs was 19.68%, exhibiting approximately a 2-fold difference (Figure 5A). In contrast, the IC_50_ values of SAL in BCSCs, MCF-7, and HUVECs were 1.66 ± 0.51 µM, 1.60 ± 0.12 µM, and 1.08 ± 0.23 µM, respectively, indicating no selective cytotoxicity for BCSCs (Figure 5B). Although API is less effective than SAL in inhibiting BCSCs, it shows superior selectivity in targeting BCSCs.

To substantiate the inhibitory effects of API on BCSCs, apoptosis was measured by flow cytometry following exposure to various API concentrations for 48 h. The results revealed that the NC group exhibited an apoptosis rate of 1.10% ± 0.13%, indicating a low level of apoptosis in BCSCs under natural conditions. In the experimental groups, treatment with API at a concentration of 7 µM led to an apoptosis rate of 8.20% ± 1.58% (*p <* 0.001, 95% CI: [5.14, 9.07]), significantly higher than NC group, demonstrating that API can effectively promote apoptosis in BCSCs even at low concentrations. As the concentration of API increased, so did the apoptosis rate, with rates of 15.41% ± 1.00% (*p <* 0.0001, 95% CI: [13.05, 15.56]) and 18.78% ± 2.26% (*p <* 0.0001, 95% CI: [14.87, 20.48]) observed at 14 μM and 28 µM concentrations, respectively, showing a clear concentration-dependent effect. Compared with the positive control drug SAL, with an apoptosis rate of 8.28% ± 0.20%, API demonstrated a stronger or at least equivalent ability to promote cell apoptosis at all tested concentrations, with the effect becoming more pronounced as the concentration increased (Figures 5C, D). These results indicate that API has a significant capacity to promote apoptosis in BCSCs, and this effect is enhanced as the concentration of API increases, showing a good concentration dependence.

### 3.5 API’s capacity to reverse the stemness of BCSCs

**FIGURE 6:**
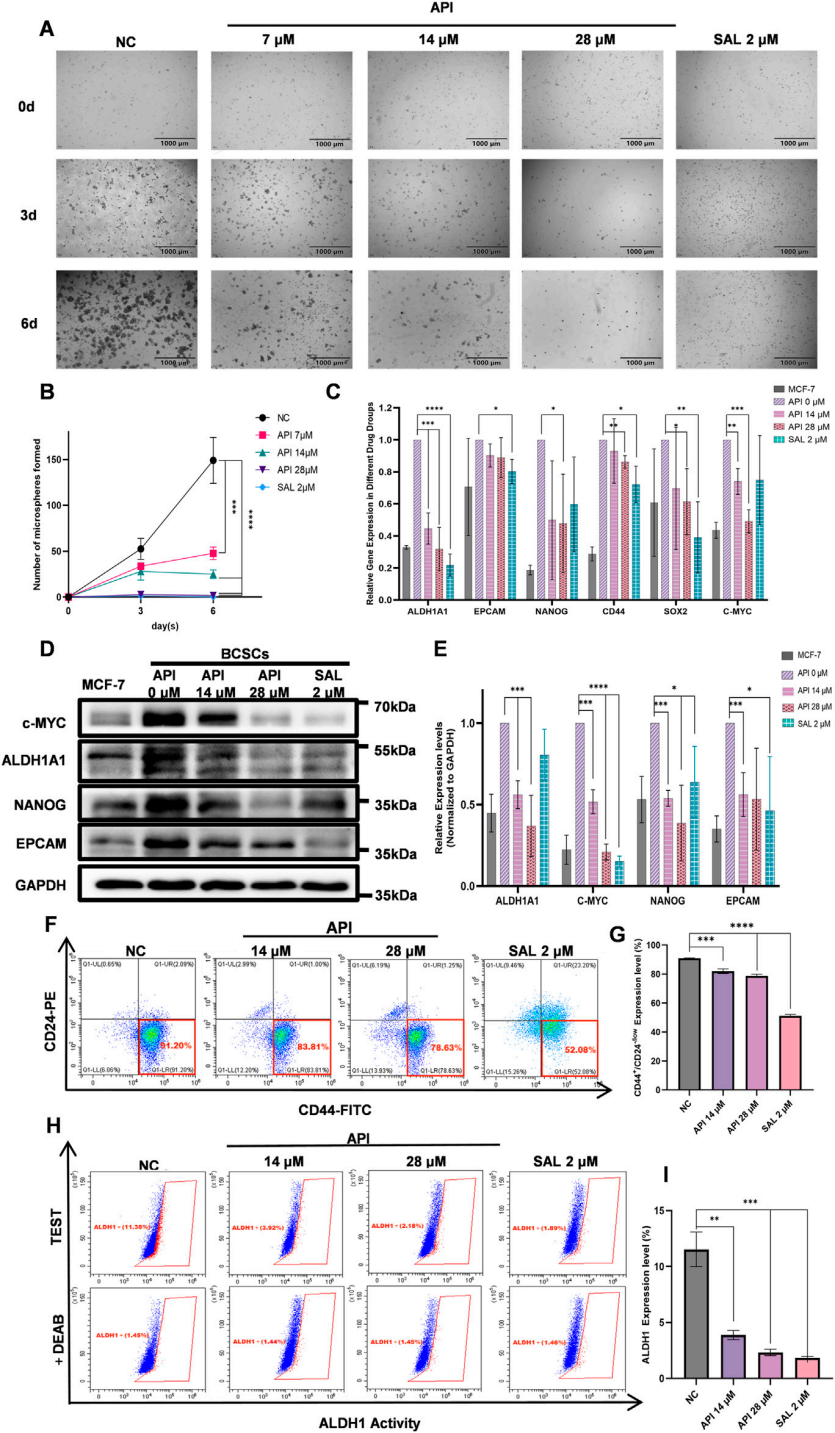
API significantly suppresses the stemness of BCSCs. **(A)** Growth and sphere-forming ability of BCSCs under API concentrations. **(B)** Sphere formation by BCSCs decreases with increasing API concentration, with a notable effect at 28 µM comparable to the positive control SAL and distinct from the untreated NC group. **(C)** Q-PCR analysis shows significant differences in the expression of stemness genes in API-treated BCSCs. **(D)** Western blot images display expression levels of stemness markers ALDH1A1, c-MYC, NANOG, and EpCAM in BCSC samples. **(E)** Quantitative data corresponding to Western blot results show. **(F)** Schematic illustration of the proportion variation of the CD44^+^/CD24^−/low^ subpopulation in BCSCs following API treatment. **(G)** Expression of the CD44^+^/CD24^−/low^ phenotype in BCSCs is significantly reduced at API concentrations of 14 and 28 µM. **(H)** Schematic presentation of the percentage changes in the ALDH1^high^ subset within BCSCs treated with different drug concentrations. **(I)** Experimental results reveal significant downregulation of ALDH1 expression in BCSCs treated with API at 14 and 28 μM “*”, *p <* 0.05; “**”, *p <* 0.01; “***”, *p <* 0.001 and “****”, *p <* 0.0001, α = 0.05.

A defining characteristic of CSCs is their capacity to form spheres. Consequently, we developed an inhibition assay to evaluate the effects of API on BCSCs. After treatment with varying concentrations of API and subsequent continuous cultivation for 6 days, we observed a significant increase in cell numbers in the NC group, primarily characterized by the aggregation of single cells into clusters and the formation of spheroids. However, with increasing concentrations of API treatment, there was a reduction in cell numbers, leading to a significant decrease in the number of observable spheroids. Additionally, the positive control drug SAL showed effectiveness in inhibiting the formation of cell spheroids without significantly affecting the total cell count. The statistical analysis demonstrated that API exerted a significant inhibitory effect on BCSCs’ formation even at low doses, with an efficacy similar to the positive control at a concentration of 28 µM (Figures 6A, B).

To further substantiate our findings, we conducted Q-PCR, Western blot, and flow cytometry analyses. The Q-PCR analysis revealed that API significantly downregulated the expression of ALDH1A1, NANOG, CD44, SRY-box transcription factor 2 (SOX2), and c-MYC. Statistical results indicated a decreasing trend in the expression levels of ALDH1A1 and c-MYC with increasing concentrations of API. Furthermore, treatment with 28 µM API led to a significant reduction in the expression levels of NANOG, CD44, and SOX2. This finding suggests that API was markedly more effective in downregulating the expression of NANOG and c-MYC compared to the SAL group (Figure 6C). Western blot analysis showed that MCF-7 cells induced to transform into BCSCs via suspension culture exhibited significantly increased expression levels of stem cell markers ALDH1A1, c-MYC, NANOG, and EpCAM. Subsequent treatment with varying concentrations of API and the positive control SAL resulted in a concentration-dependent decrease in the expression of these markers. Statistical analysis revealed that API significantly downregulated the expression of ALDH1A1 and NANOG at both tested concentrations, with greater efficacy than the SAL group. Additionally, API demonstrated a significant reduction in the protein expression of c-MYC and EpCAM, particularly in the inhibition of c-MYC expression (Figures 6D, E). Consistent with the Western blot data, flow cytometry analysis revealed that the inhibitory effect on BCSCs increased with higher concentrations of API after a 48-h treatment. In the NC group, the percentage of CD44^+^/CD24^−/low^ cells was 90.93% ± 0.29%. After treatment with 14 μM and 28 µM API, the percentages of CD44^+^/CD24^−/low^ cells were reduced to 81.66% ± 1.64% (*p <* 0.001, 95% CI: [6.34, 11.52]) and 78.76% ± 1.18% (*p <* 0.0001, 95% CI: [10.21, 14.11]), respectively, demonstrating significant differences. The percentage of ALDH1^high^ cells also decreased from 11.54% ± 1.55% in the NC group to 3.88% ± 0.41% (*p <* 0.01, 95% CI: [5.09, 10.00]) and 2.32% ± 0.28% (*p <* 0.001, 95% CI: [6.70, 11.74]) after treatment with 14 μM and 28 µM API, respectively, also showing significant differences (Figures 6F–I). Collectively, these findings indicate that API effectively reduced the formation of BCSCs.

### 3.6 API reduces stemness of BCSCs through PI3K/AKT/p53 signaling pathway

**FIGURE 7:**
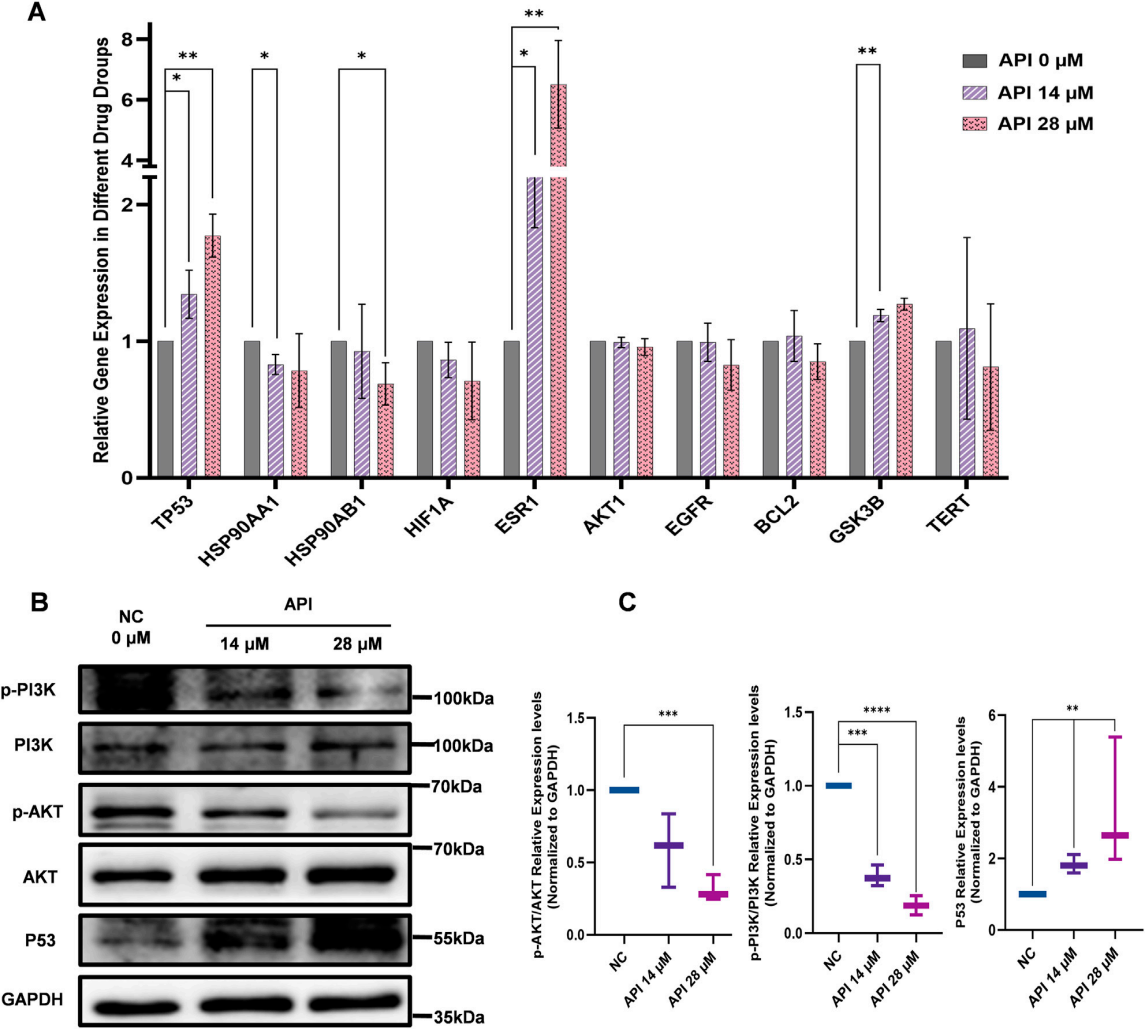
API reverses stemness, inhibits proliferation, and promotes apoptosis in BCSCs via the PI3K/AKT/p53 Pathway. **(A)** Q-PCR analysis indicates significant changes in the expression of top ten MCC-scored genes post-API treatment, with notable variation in TP53 expression. **(B)** Western blot analysis of pathway markers in BCSCs, Including p53, AKT, p-PI3K, PI3K, p-AKT, and AKT. **(C)** Quantitative analysis of protein expression levels of p-PI3K/PI3K, p-AKT/AKT, and p53 in BCSCs. “*”, *p <* 0.05; “**”, *p <* 0.01; “***”, *p <* 0.001 and “****”, *p <* 0.0001, α = 0.05.

The results of network pharmacology and molecular docking analysis indicate potential interactions between API with TP53 and the PI3K/AKT signaling pathways, and further investigations of BCSCs were performed using Q-PCR and Western blot. These findings substantiate the hypothesis that the PI3K/AKT/p53 signaling pathway is instrumental in mediating the effects of API on BCSCs. To delineate the molecular mechanisms underlying API’s inhibitory effects on BCSCs, we assessed the mRNA levels of the top 10 genes within our PPI network. Q-PCR data revealed that treatment with 14 µM or 28 µM API for 48 h resulted in a significant upregulation of TP53 and ESR1 expression in enriched BCSCs (Figure 7A). The p53 protein, a pivotal tumor suppressor, is integral to the regulation of the cell cycle, the induction of apoptosis, and the repair of damaged DNA, as evidenced by literature (Boutelle and Attardi, 2021; Rodriguez-Pastrana et al., 2023; Wang et al., 2023). In cancer therapy, the PI3K/AKT signaling pathway is often targeted to inhibit cancer cell proliferation (He et al., 2021). Moreover, the PI3K/AKT signaling pathway can indirectly regulate TP53 expression through MDM2 proto-oncogene (MDM2)-mediated pathways (Abraham and O’Neill, 2014). Therefore, based on previous pathway analysis, we chose to further investigate the TP53 and PI3K/AKT signaling pathways at the protein level. Western blot analysis revealed that with increasing concentrations of API, the relative expression levels of p-PI3K and p-AKT proteins gradually decreased, while the expression level of TP53 protein significantly increased with API concentration. These results indicate that API modulates the PI3K/AKT/TP53 signaling pathway by downregulating the phosphorylation levels of PI3K and AKT and upregulating TP53 expression, thereby exerting its inhibitory effect on BCSCs (Figures 7B, C).

## 4 Discussion

In this study, we aimed to investigate the therapeutic potential of API, a compound known for its high safety profile and significant anticancer activity. Extensive research has supported the efficacy of API in cancer treatment (Parajuli et al., 2009). The combination of API with oxaliplatin exerts a significant impact on non-small cell lung cancer (NSCLC) by modulating the EGFR and its downstream signaling pathways (Ji et al., 2023). Furthermore, the synergistic combination therapy of API with sorafenib has been demonstrated to significantly enhance the antitumor efficacy against hepatocellular carcinoma (Singh et al., 2024). Additionally, the combination therapy of API and vorinostat induces apoptosis-mediated cell death in TNBC by modulating epigenetic modifications, apoptotic regulators, and associated miRNAs (Nimal et al., 2024). Although API shows promise as a candidate for combination therapy to improve therapeutic outcomes, there remains a research gap in the context of targeting BCSCs in breast cancer treatment. Breast cancer, the second most prevalent and fatal malignancy among women, has experienced a significant increase in incidence over the past four decades (Nimbannavar and Mane, 2019). Despite medical technological advancements, the prognosis and the incidence of adverse drug reactions associated with breast cancer treatment continue to pose significant challenges. CSCs, a subclass of malignant cells within tumors, are increasingly recognized as playing a role in tumor initiation, therapeutic escape, and recurrence (Li et al., 2022; Ray and Mukherjee, 2024). As a subset of CSCs, BCSCs are particularly resistant to conventional therapies, which significantly contributes to the limited success of treatments.

ALDH1A1 is an enzyme that plays a crucial role in various cancers, particularly in breast cancer, where it activates the TAK1-NFkB signaling pathway by reducing the intracellular pH of tumor cells, increases the secretion of GM-CSF, promotes the proliferation of myeloid-derived suppressor cells (MDSCs), inhibits the activity of CD8^+^ T cells, and thus promotes tumor growth (Liu et al., 2021). NANOG expression is closely associated with the stem cell characteristics, invasiveness, and apoptosis of breast cancer, and reducing its expression can decrease the proportion of BCSCs and their self-renewal capacity (Arif et al., 2015). C-MYC, an important transcription factor, regulates apoptosis by modulating the expression of pro-apoptotic and anti-apoptotic proteins (Arif et al., 2015). Desialylated EPCAM promotes apoptosis in breast cancer cells by activating the PI3K/AKT/mTOR signaling pathway (Pandkar et al., 2023). CD44^+^/CD24^−/low^, like ALDH1^high^, as markers of BCSCs, possess the ability to self-renew and form tumors, playing a vital role in the progression and metastasis of breast cancer (de Beça et al., 2013; Gyan et al., 2021). The expression levels of these genes or proteins not only reflect the ability of candidate drugs to reduce cellular stemness but also demonstrate their effectiveness in inhibiting cancer cell proliferation and promoting apoptosis.

**FIGURE 8:**
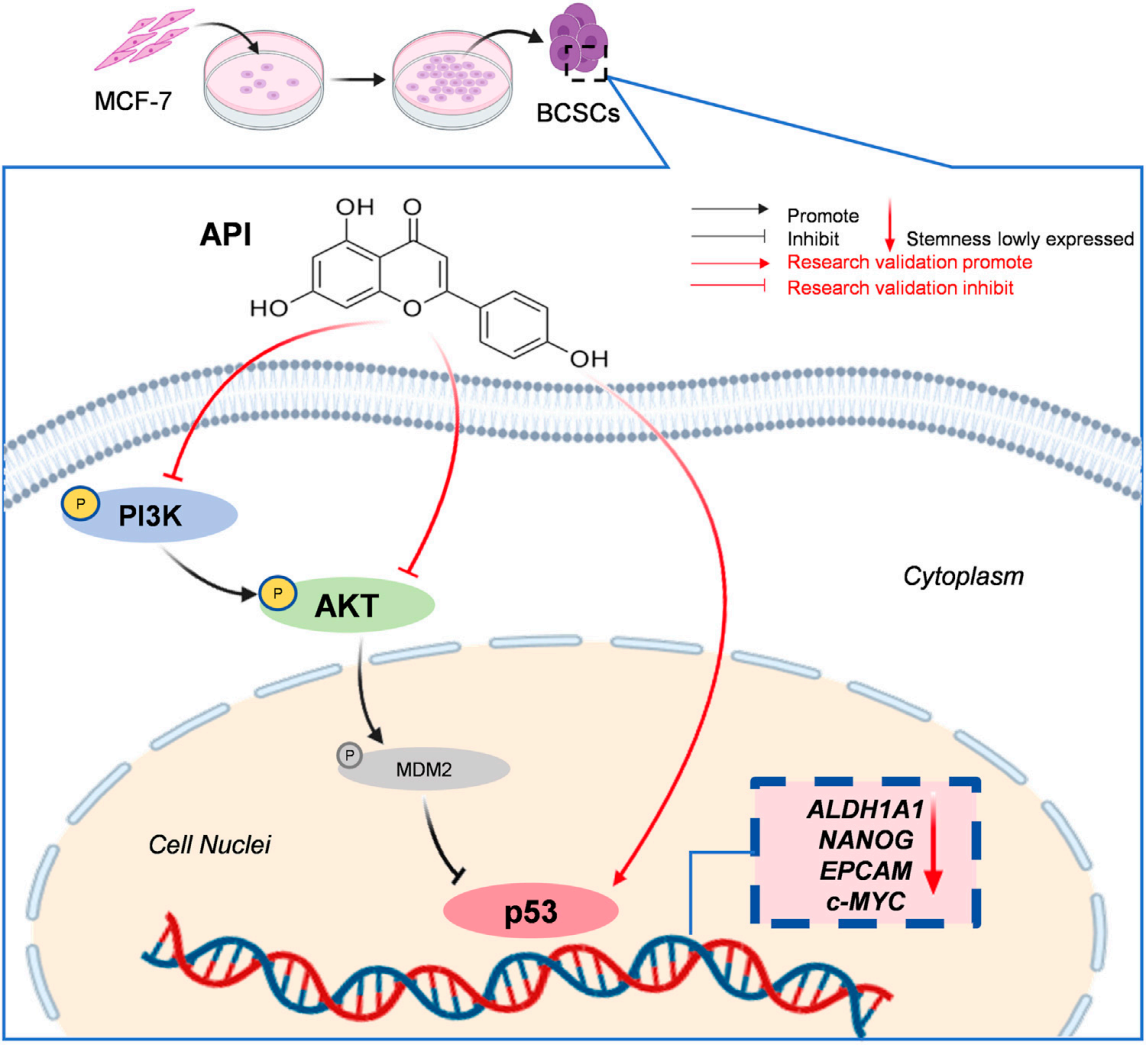
The mechanism of API against BCSCs via PI3K/AKT/p53 pathway.

Our study, integrating network pharmacology with *in vitro* assays, demonstrates that API exerts its therapeutic effects on BCSCs by modulating the PI3K/AKT/p53 signaling axis (Figure 8). AKT, a central kinase in the PI3K/AKT pathway, is implicated in various cellular processes, including those regulated by the p53 pathway, cell proliferation, apoptosis, and cell cycle control (Chen et al., 2023). AKT is capable of phosphorylating and thereby inactivating p53, either directly or indirectly through modulation of MDM2, an E3 ubiquitin ligase responsible for p53 degradation. The nuclear phosphoprotein p53 serves as a downstream target of the PI3K/AKT pathway (Zhang J. et al., 2023) and is widely recognized as a tumor suppressor, regulating critical cellular functions such as the cell cycle, apoptosis, and stemness properties (Chen, 2016; Hermawan et al., 2021). Evidence suggests that p53 functions as a barrier to the formation of cancer stem cells and is downregulated in mammospheres (Aloni-Grinstein et al., 2014). The expression and functionality of p53 are frequently compromised in various cancers, highlighting its essential role in maintaining the stemness characteristics of cancer cells (Shetzer et al., 2014; Hermawan et al., 2021). Upon p53 inactivation by AKT, it loses its ability to regulate these processes effectively, leading to evasion of apoptosis and uncontrolled cell proliferation, which contribute to CSC maintenance and tumorigenesis. Studies have shown that mutations activating the PI3K/AKT signaling pathway, along with the inactivation of the TP53 tumor suppressor gene, are common mechanisms for cancer cell proliferation and evasion of programmed cell death. Furthermore, evidence has accumulated that, under certain conditions, the PTEN/PI3K/AKT signaling pathway may also positively regulate p53 by enhancing its translation and protein stability, implying the involvement of additional mechanisms in AKT-mediated p53 regulation (Abraham and O’Neill, 2014). These studies demonstrate that the PI3K/AKT/p53 signaling pathway plays a pivotal role in the development of cancer (Zhang J. et al., 2023; Ning et al., 2024; Zheng et al., 2024). Thus, interrupting this signaling axis presents a promising therapeutic approach for anti-cancer strategies.

Molecular docking and subsequent Western blot analysis have confirmed the interaction of API with key proteins within the PI3K/AKT/p53 pathway, thereby validating its capacity to inhibit BCSC functions. Significantly, the data reveal a concentration-dependent decrease in BCSC stemness markers, such as ALDH1A1, NANOG, c-MYC, and EpCAM, following API treatment, as evidenced by flow cytometry and Western blot. Our findings demonstrate that API effectively suppresses BCSC proliferation, enhances apoptosis, and reduces stemness properties through modulation of the PI3K/AKT/p53 signaling pathway. Notably, API markedly reduced the phosphorylation of PI3K and AKT (*p <* 0.001, *p <* 0.0001, α = 0.05), suggesting an inhibition of PI3K/AKT activation, while concurrently increasing the expression of p53 (*p <* 0.01, α = 0.05), potentially restoring p53-mediated tumor suppression mechanisms (Ghosh et al., 2023; Yang et al., 2023; Nakagawa-Saito et al., 2024).

This study, therefore, offers fresh perspectives on the multifaceted nature of API’s anti-cancer actions, consistent with recent reports that underscore its ability to suppress stem cell-like characteristics in triple-negative breast cancer cells by inhibiting YAP/TAZ activity (Li et al., 2018) and its influence on programmed death ligand 1 expression (Coombs et al., 2016).

The findings have significant implications, emphasizing the need to understand the precise molecular targets and pathways influenced by API in BCSCs. Elucidating the role of API in the PI3K/AKT/p53 pathway provides a scientific foundation for future preclinical and clinical investigations intended to harness this natural compound for the targeted elimination of BCSCs and to improve patient outcomes in breast cancer treatment. The effective modulation of BCSC self-renewal and differentiation by API may facilitate the development of novel, safer, and more efficacious therapeutic strategies to counter breast cancer recurrence and metastasis.

## 5 Conclusion

This study has provided evidence for the therapeutic potential of API in targeting BCSCs, a subpopulation of breast cancer cells that are known to contribute significantly to tumor initiation, progression, and recurrence. Through a comprehensive approach that integrated network pharmacology, molecular docking, and experimental validation, we have elucidated the molecular mechanisms underlying the anti-BCSC effects of API. Our findings demonstrate that API exerts its inhibitory effects on BCSCs, particularly through its impact on the PI3K/AKT/p53 signaling pathway.

## Data Availability

The original contributions presented in the study are included in the article/supplementary material, further inquiries can be directed to the corresponding authors.
